# The Efficacy of Virtual Reality Training in the Rehabilitation of Orthopedic Ankle Injuries: A Systematic Review and Meta-analysis

**DOI:** 10.1177/11795727231151636

**Published:** 2023-02-07

**Authors:** Abd El Rahman Elaraby, Mostafa Shahien, Alhadi M. Jahan, Mohammad Etoom, Amira Hassan Bekhet

**Affiliations:** 1 Faculty of Physical Therapy, Cairo University, Giza, Egypt; 2 Impact Research Group, Cairo, Egypt; 3 School of Rehabilitation Sciences, University of Ottawa, Ottawa, ON, Canada; 4 Department of Physiotherapy, College of Medical Technology, Misrata, Libya; 5 Division of physical therapy, allied medical sciences department, Aqaba University of Technology, Aqaba, Jordan; 6 Medical Research Group of Egypt (MRGE), Cairo, Egypt

**Keywords:** Ankle, functional ankle instability (FAI), balance, ankle sprain, virtual reality, rehabilitation

## Abstract

**Introduction:**

Orthopedic ankle injuries are considered among the most common musculoskeletal injuries. A wide variety of modalities and techniques have been used for the management of these injuries, and virtual reality (VR) is one modality that has been examined in ankle injuries rehabilitation.

**Purpose:**

This study aims to systemically review previous studies evaluating the effect of virtual reality in rehabilitating orthopedic ankle injuries.

**Methods:**

We searched six online databases: PubMed, Web of Science (WOS), Scopus, the Physiotherapy Evidence Database (PEDro), Virtual Health Library (VHL), and Cochrane Central Register of Controlled Trials (CENTRAL).

**Results:**

Ten randomized clinical trials met the inclusion criteria. Our results showed that VR had a significant effect on overall balance compared to conventional physiotherapy (SMD = 0.359, 0.009–0.710 *P* = 0.04), [*I* ^2^= 17%, *P* = 0.30]. Compared with conventional physiotherapy, VR programs significantly improved gait parameters such as speed and cadence, muscle power, and perceived ankle instability; however, no significant difference was detected in the foot and ankle ability measure (FAAM). Additionally, significant improvements in static balance and perceived ankle instability were reported after the use of VR balance and strengthening programs. Finally, only two articles were deemed to have good quality, and the other studies’ quality ranged from poor to fair.

**Conclusion:**

VR rehabilitation programs can be used to rehabilitate ankle injuries, as they are regarded as safe interventions and have promising effects. However, there is a need for studies with high quality since most included studies’ quality varied from poor to fair.

## Introduction

Orthopedic ankle injuries involve injuries of many types, such as lateral ankle sprains (LAS) or functional ankle instability (FAI). Lateral ankle sprains are prevalent among adults and athletes participating in sports that require jumping and frequent changes in direction.^1^ Yearly, two million individuals injure their ankles in the US and UK, accounting between 3% and 5% of emergency department visits, and over 700 individuals worldwide suffer LAS daily.^2,3^ The involuntary twisting of the joint during a sprain has a significant impact on static and dynamic balance, muscle reaction time, muscle strength, and joint proprioception, leaving a painful and swollen joint with limited function.^4,5^ Those factors combined with insufficient or untimely rehabilitation after the initial injury pose a high risk for re-injury, leading to FAI or chronic ankle instability (CAI).^6^ FAI produces a persistent feeling of giving way or instability within the joint during normal daily activities and affects about 70% of LAS patients.^7,8^ LAI and FAI affect normal biomechanical alignment and body-weight bearing, causing long-term disability, and chronic debilitating health consequences such as osteoarthritis and low quality of life, posing a high socioeconomic burden.^9^

According to the 2016 National Institute for Health And Care Excellence (NICE) guidelines, PRICE, an acronym standing for Protection, Rest, Ice, Compression, and Elevation, is the main protocol for dealing with acute ankle injuries and is associated with the early administration of pain-controlled exercises, manual therapy, muscle strength, and balance exercises.^10^ Strict adherence to those rehabilitation strategies can prevent the occurrence of FAI.^11^ Despite the evident effects of conventional therapy on strength, balance, and other clinical outcomes, it has the disadvantage of being boring, affecting the patients’ compliance, aggravating the condition, and posing a high economic burden.^12^ Therefore, ankle rehabilitation has recently encompassed new trends like virtual reality (VR) rehabilitation modalities.

The concept of VR was first developed by Ivan Sutherland in 1960.^13^ VR systems offer a window of interaction between users and a computer environment simulating real-life.^13^ VR systems can be either immersive or non-immersive, according to the user's level of interaction with the virtual environment and the number of stimulated senses.^13^ VR was first adopted in the healthcare field during the 1990s to present compound medical data, especially when planning for surgery.^14^ Since then, the use of VR in the healthcare field has grown exponentially to encompass many fields, such as teaching, training, and rehabilitation.^14^ Over the last decade, VR use in rehabilitation has surged thanks to its three elements, Interaction, Immersion, and Imagination, which could profoundly enhance motor learning.^13,14^ VR provides some advantages over conventional therapy. It provides a virtual environment with functional tasks and immediate feedback with proper incentives to encourage patients to increase adherence, and also offers personalized programs for each patient and motivates them to try risky challenges within a safe virtual environment, boosting their capabilities. VR can be used as a home-based program with endless repetitions, thus alleviating work stress for physical therapists, as it requires only mild supervision.^15,16^

There is an abundance of literature investigating the role of VR in neuro-rehabilitation that shows promising results in patients with stroke, multiple sclerosis, and Parkinsonism.^17-19^

Gumaa and Yussef^15^ conducted a systematic review to evaluate the efficacy of virtual reality in all orthopedic conditions. For ankle injuries, they reviewed only four studies that included participants with lateral ankle sprain and reported mixed results. Due to the small number of clinical trials they included for the ankle injury, they could not perform meta-analysis. It is evident that there is a need to expand on Gumaa and Yussef’s work and provide the best available evidence in rehabilitating ankle injuries using Virtual Reality systems. Therefore, this study aims to synthesize the evidence for the effect of VR specifically in patients with orthopedic ankle injuries.

## Methods

### Literature search strategy

This study was registered through PROSPERO, the International prospective register of systematic reviews (CRD42021230879). We conducted and reported this review in accordance with the PRISMA 2020 (the Preferred Reporting Items for Systematic Reviews and Meta-Analyses guidelines).^20^

Six databases were searched; namely, Pubmed, Web of Science (WOS), Scopus, the Physiotherapy Evidence Database (PEDro), Virtual health library (VHL), and Cochrane Central Register of Controlled Trials (CENTRAL) from inception till May 17, 2022. The search was conducted using relevant keywords; “virtual,” “virtual reality,” “virtual environment,” “VR content,” “virtual rehabilitation,” “computer-based,” “computer-interface,” “cyberspace,” “artificial intelligence,” “computer simulat*,” “simulator,” “exergam*,” “active video gam*,” “interactive gam*,” “game,” “gaming,” “gamification,” “Xbox Kinect,” “X-box,” “Kinect,” “Nintendo,” “Wii,” “Nintendo Wii,” “ski simulation,” “augmented reality,” “ankle,” “physical therapy,” “physiotherapy,” “training,” “rehabilitation,” “exercise,” “intervention.” We compiled the former keywords using Boolean operators and adjusted the search strategy according to each database. Filters were applied to limit the retrieved studies to English articles with human participants. In addition, we manually searched the reference lists of the included studies to detect any relevant studies.

### Eligibility criteria

We set the selection criteria using PIOCS (P-population, I-intervention, C-comparison, O-outcome, S-study design). Included only English randomized controlled trials that (1) recruited adults (⩾18 years old) with orthopedic ankle injuries, (2) used virtual reality rehabilitation techniques either alone or as adjuvant therapy, (3) compared various programs of virtual reality programs or using conventional physical therapy, placebo, or no intervention for the control group, and (4) used any outcome to measure the effect of the intervention. We excluded articles that (1) included patients with neurological disorders and (2) used virtual reality techniques for any other use rather than motor rehabilitation.

### Study selection

Two reviewers independently screened the titles and abstracts of the retrieved articles using predetermined eligibility criteria. Any disagreements or discrepancies were resolved by a third reviewer until consensus was reached.

### Data extraction

The full texts of the included articles were further analyzed. The following data were extracted: sample size, participant’s age and gender, type, dose of intervention, virtual reality device, diagnosis, outcome measures, and the main results. Any potential conflicts were resolved by a third reviewer.

### Quality appraisal

The methodological quality of the included studies was independently assessed by two reviewers using the modified Downs and Black scale for clinical trials.^21^ The scale consists of 27 questions rating four categories: (1) reporting, (2) external validity, (3) internal validity, and (4) power. Studies are considered of excellent quality when the final score ranges from 26 to 28, good quality if the score ranges from 20 to 25, fair quality if the score ranges from 19 to 15, and poor if the score is 14 or less. Any disagreements or discrepancies were resolved by discussion till a consensus was reached. The Downs and Black checklist was chosen based on several factors. For example, the checklist has good psychometric properties, such as internal consistency, test–retest reliability, inter-rater reliability, and criterion validity.^21^ Importantly, the checklist is a universally accepted tool in evaluating the methodological quality of studies as it provides more interpretable scores, which is essential when completing a systematic review and meta-analysis of intervention studies.

### Data synthesis and analysis

Meta-analysis was conducted if at least two studies compared the efficacy of two different programs of VR or examined the effect of VR against traditional intervention, no intervention, or placebo. Standardized mean difference (SMD), 95% confidence interval (CI), and *P* value were calculated by comparing changes in the outcomes between the VR and the control groups using the random-effect model of analysis.^22^ That is, SMD values of 0.2–0.5 are considered small effect size, 0.5–0.8 medium effect size, and >0.8 are considered large effect size. Effect size tells how meaningful the relationship between variables or the difference between groups is. A large effect size means that a research finding has clinical significance, while a small effect size indicates limited clinical applications. In other words, in the context of this study the larger the effect size the stronger the relationship between the variables. Heterogeneity in treatment effect was examined by calculating the *I*^2^ index. The level of significance was set at a *P*-value of up to 0.05. All meta-analyses were carried out using the comprehensive meta-analysis, version 2.2.064 software package (Biostat, Englewood, New Jersey, USA).

## Results

### Study selection

**Figure 1.**
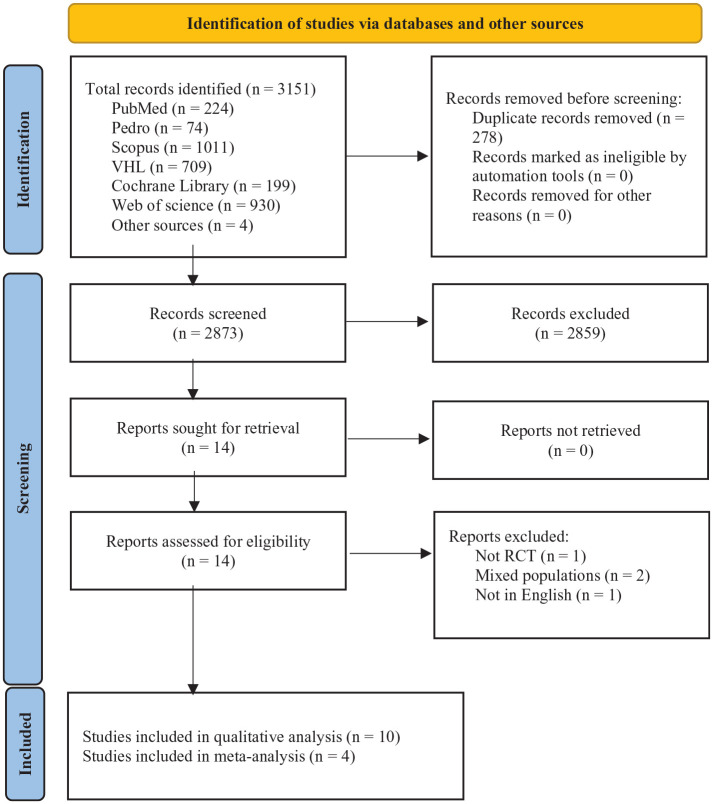
PRISMA flow diagram of the screening and study selection process.

Figure 1 represents the flow of the selection process during the study. We conducted an electronic search of six online databases from inception till May 17, 2022. The search strategy retrieved 224 records from PubMed, 74 from PEDro, 1011 from Scopus, 709 from the virtual health library, 199 from the Cochrane library, and 930 from Web of Science. After removing 278 duplicates and including additional 4 RCTs obtained via a manual search, we screened the titles and abstracts of 2873 records. Fourteen articles underwent full-text screening after excluding 2859 records. Two articles^23,24^ used a mixed population of ankle and other lower-limb orthopedic injuries, and so we contacted their authors for the data of the participants with orthopedic ankle injuries, but no response was received. Thus, we also excluded those two articles from the analysis. In addition, two other studies^25^ were excluded due to an inappropriate research design and using a language other than English. Finally, ten RCTs were included in the qualitative analysis,^26-35^ and four were included in the meta-analysis.^27,31,32,34^

### Quality assessment

All ten articles were assessed against the Downs and Black checklist. Four studies^29,30,32,34^ had a poor quality rating, four^26-28,31^ achieved a fair quality rating, and the other two articles^33,35^ were deemed to be of good quality. The main areas of weakness in the ten studies were in terms of external validity and the power analysis of the Modified Downs and Black scale. Reporting was good among the 10 studies. Finally, internal validity showed a high quality only in two studies.^33,35^ The complete quality assessment scores can be found in Supplemental file (1).

### Characteristics of the included studies

Collectively, the studies included 394 participants diagnosed with functional ankle instability^26-32^ and lateral ankle sprain.^33,35^ The participants’ ages ranged from 16 to 44 years old, and the ten studies were conducted in three countries: seven in South Korea,^26-32^ two in Switzerland,^33,35^ and one in Greece.^12^

The included studies used various forms and types of VR to manage orthopedic ankle injuries. Eight studies^26-31,33,35^ used the Nintendo Wii Fit Plus device to improve muscle strength and balance. For muscle strength, six studies^26-31^ used lunges, single leg extensions, sideways leg lifts, single leg twists, and rowing squats, and for balance, six studies^27-31,35^ used ski slalom, table tilt soccer heading, tightrope walking, and snowboard slalom, while the other two studies^33,35^ added the penguin slide and balance bubble to the previous exercises. An XBK device was also used by one study^34^ as a virtual reality method for balance training by playing XbK games such as Rally Ball, Reflex Ridge, River Rush, and 2000 Leaks. Only one study^32^ used a visual feedback balance trainer device for balance training in individuals with FAI.

Seven studies^26,27,31-35^ compared the efficacy of VR, and investigated its influence on balance using the Biodex Balance System and Biorescue, spatiotemporal gait parameters, on muscle strength using a Biodex isokinetic dynamometer, on physical function using the foot and ankle ability measure (FAAM), on pain using a visual analogue scale (VAS), and on ankle instability using the Cumberland Ankle Instability Tool (CAIT). Three studies^28-30^ investigated the efficacy of two VR programs; namely, a balance training VR program and a strength training VR program, and compared their effect on balance using the Biodex Balance System, on muscle strength and ankle proprioception using a Biodex isokinetic dynamometer, and on ankle instability using CAIT. Detailed descriptions of study characteristics, interventions, outcomes, and the main results are given in Tables 1 and 2.

**Table 1.** Characteristics of the included studies.

**Author(s)**	**Country**	**Design**	**Sample size**	**Age in years (Mean ± SD)**	**Diagnosis**	**VR type**	**QA**
Kim and Heo^31^	Korea	RCT	21	21 ± 1.2	FAI	Nintendo Wii Fit Plus	17
Punt et al^33^	Switzerland	RCT	90	Intervention 1 (34.7 ± 10.7) Intervention 2 (34.7 ± 11.3) Control (33.5 ± 9.5)	LAS; grade I or II	Home based Nintendo Wii Fit Plus	20
Kim et al^26^	South Korea	RCT	20	Intervention 1 (21.8 ± 1.2) Intervention 2 (22.1 ± 2.4)	FAI	Nintendo Wii Fit Plus	15
Kim and Jun^28^	South Korea	RCT	20	23.3 ± 2.4	FAI	Nintendo Wii Fit Plus	13
Punt et al^35^	Switzerland	RCT	90	Intervention 1 (34.7 ± 10.7) Intervention 2 (34.7 ± 11.3) Control (33.5 ± 9.5)	LAS; grade I or II	Home based Nintendo Wii Fit Plus	20
Nam et al^32^	South Korea	RCT	28	Intervention 1(23.0 ± 2.9), Control (23.7 ± 1.8)	FAI	Bal Pro (Man &Tel Co., Korea)	13
Kim and Heo^30^	South Korea	RCT	20	23.3 ± 2.4	FAI	Nintendo Wii Fit Plus	14
Vernadakis et al^34^	Greece	RCT	63	16 ± 1	LAS	XBK	14
Kim and Gang^27^	South Korea	RCT	22	21.6 ± 2.3	FAI	Nintendo Wii Fit Plus	15
Kim et al^29^	South Korea	RCT	20	Intervention 1 (22.6 ± 1.4) Intervention 2 (23.2 ± 1.0)	FAI	Nintendo Wii Fit Plus	15

Abbreviations: FAI, functional ankle instability; LAS, lateral ankle sprain; QA, quality assessment; RCT, Randomized controlled trial; XBk, Xbox Kinect.

**Table 2.** Summary of the interventions, outcomes, and the main results.

**Author(s)**	**Intervention group 1**	**Intervention group 2**	**Control group**	**Dosage**	**Outcome measures**	**Results**
**Kim and Heo** ^31^	Strengthening and balance VR programs	Strengthening and balance exercises	–	20 min/d, 3 d/wk. for 4 wks.	Balance using Biodex Balance System	Static balance and dynamic balance were significantly lower in the overall direction than conventional exercise.
**Punt et al** ^33^	Wii Fit™ balance games	Conventional rehabilitation	NO exercise at all	E1 (30 min/d, 2 d/wk. for 6 wks), E2 (30 min/day, 9 d for 6 wk.)	• Gait speed • Cadence • Step length • Single-support time • Symmetry index of the step length • Symmetry of the single-support time • Maximum dorsiflexion and maximum plantar flexion	Gait speed, cadence and step length improved significantly in the three groups. The single support time and symmetry index of step length improved significantly in the Wii Fit™ group.
**Kim et al** ^26^	Strengthening and balance VR programs	Strengthening and balance exercises	–	20 min/d, 3 d/wk. for 4 wks.	Ankle muscle strength using Biodex isokinetic dynamometer	VR group showed significant improvement only for PF, while the TRI group showed significant improvements in the 4 ankle motions. No significant differences were found between the 2 groups.
**Kim and Jun** ^28^	VR strengthening exercises	VR balance programs		20 min	Ankle muscle strength using Biodex isokinetic dynamometer	For strengthening group, only PF and DF showed statistically significant improvement. In the balance group, the 4 ankle motions showed statistically significant improvement.
**Punt et al** ^35^	Wii Fit™ balance games	Conventional rehabilitation	NO exercise at all	E1 (30 min/d, 2 d/wk. for 6 weeks), E2 (30 min/day, 9 d for 6 wk.)	• Self-reported physical function using FAAM • Pain at rest and while walking using VAS	FAA showed significant improvement in all groups. VAS scores during rest and walking showed significant improvement in the Wii Fit™ group. No significant differences were detected regarding VAS scores among the 3 groups.
**Nam et al** ^32^	Visual feedback balance training and ankle joint exercises	stretching and muscle-strengthening exercises	–	30 min/d, 3 d/wk. for 8 wks.	• Balance using Biorescue to measure LOS • Ankle instability using CAIT	LOS measurements showed significant improvement in the experimental group. Both groups showed significant improvement in terms of CAIT scores.
**Kim and Heo** ^30^	VR strengthening exercises	VR balance programs	–	20 min/d, 3 d/wk. for 4 wks.	Balance using Biodex Balance System	Intervention group 1 showed significant improvement regarding static balance in the ML direction and the overall dynamic balance. Intervention group 2 showed significant improvement in the 3 directions of static balance and overall and AP dynamic balance.
**Vernadakis et al** ^34^	XbK balance games	Mini trampoline and inflatable discs exercises	NO exercise at all	24 min/d, 2 d/wk. for 10 wks.	Balance using Biodex Stability System	OSI and LOS scores showed significant improvement for both experimental groups but showed no improvement in the control group.
**Kim and Gang** ^27^	Ankle kinesio tapping accompanied with VR strengthening and balance programs.	-	No exercise at all	20 min/d, 3 d/wk. for 4 wks.	Balance using Biodex balance system	The 3 dynamic balance indexes showed significant improvement in the interventional group, while the 3 static balance indexes showed no improvement.
**Kim et al** ^29^	VR strengthening exercises	VR balance programs	–	20 min/d, 3 d/wk. for 4 wks.	• Ankle proprioception using Biodex isokinetic Dynamometer • Ankle Instability using CAIT	Proprioception in the sagittal and horizontal planes showed significant improvement in experimental group 2 group but no improvement in experimental group 1. CAIT scores showed significant improvement in both groups.

Abbreviations: AP, anterior-posterior; CAIT, Cumberland ankle instability tool; d, day; DF, dorsiflexion DF; FAA, foot and ankle ability; FAAM, foot and ankle ability measure; LOS, limit of stability; min, minutes; ML, medio-lateral; OSI, overall stability index; PF, plantar flexion; TRI, traditional rehabilitation interventions; VAS, visual analogue scale; VR, virtual reality; wk, week; XBk, Xbox Kinect.

## Effect of VR on study outcomes

### Efficacy of VR compared to conventional physiotherapy or no treatment

**Figure 2.**
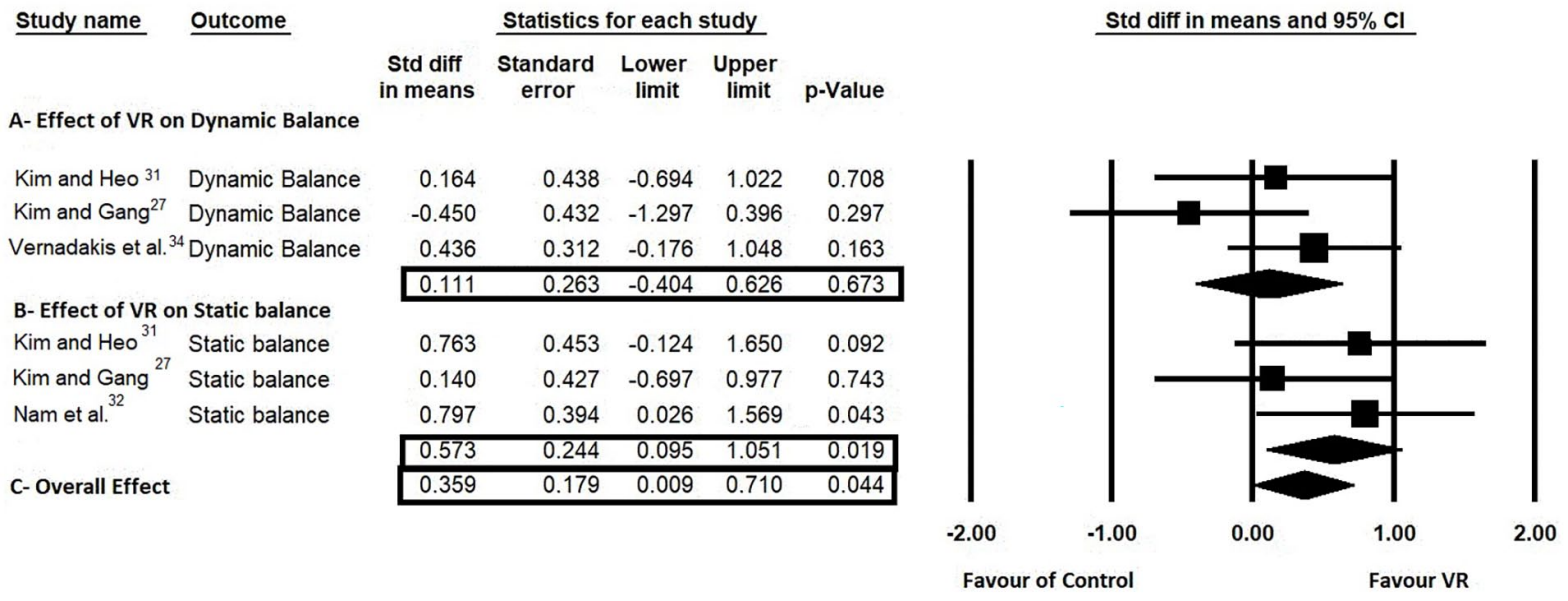
Forest plots of mean difference comparing the efficacy of VR compared to conventional physiotherapy or no treatment regarding dynamic and static balance.

Regarding balance, we pooled the results of four studies, and the meta-analysis showed a significant and homogeneous effect of VR on overall balance (SMD = 0.359, 0.009–0.710, *P* = 0.04), [*I*^2^ = 17. %, *P* = 0.30], (Figure 2). Subgroup analysis was conducted to investigate the efficacy of VR on static and dynamic balance, and it was concluded that VR significantly improves static balance (SMD = 0.573, 0.095–1.051, *P* = 0.02), [*I*^2^ = 7%, *P* = 0.47], while having no significant effect on dynamic balance (SMD = 0.111, −0.404 to 0.628, *P* = 0.67), [*I*^2^ = 28%, *P* = 0.25], Figure (2).

Punt et al^33^ examined the effect of VR on spatiotemporal gait parameters, and concluded that VR significantly improved gait speed, cadence, step length, single support time, and the symmetry index of single support time. VR did not improve the movement or timing of dorsiflexion or plantarflexion during the swing phase, and there was no significant improvement from VR on spatiotemporal gait parameters in comparison with the conventional physical therapy group or the no-intervention group.^33^

Regarding muscle power, Kim et al^26^ reported no significant effect from VR on ankle muscle power compared with the traditional exercise group. Regarding perceived ankle stability, using CAIT, Kim and Jun^28^ reported a significant improvement from VR. Punt et al^35^ reported a significant improvement in FAAM in the VR group; however, no superior effect from VR was reported in comparison with the traditional physiotherapy or no-treatment groups.

### Efficacy of VR for balance program compared to VR for strengthening program

Kim and Heo^30^ investigated the effectiveness of balance VR programs compared to strengthening VR programs on static and dynamic balance, and reported that both programs effectively improved static and dynamic balance.

Regarding muscle power, the results of one study^35^ showed better results in favor of the VR balance training program. Another study^28^ found that the VR balance training program improved ankle proprioception, while there was no effect from the VR strengthening training program. Nam et al^32^ reported that the different VR programs significantly improved perceived ankle stability as measured by CAIT.

### Adverse events and side effects

None of the included studies reported any adverse events or side effects from the virtual reality interventions.

## Discussion

### Main findings

This systematic review and meta-analysis verified the efficacy of Virtual Reality (VR) training for individuals with ankle joint injuries. A total of ten RCTs were included in the qualitative analysis; however, it was only possible to compare the pooled effects of VR training in relation to dynamic balance and static balance in four studies.

The quality of the included studies was generally moderate, with two studies demonstrating good methodological quality on the Downs and Black scale.^21^ In terms of the studied outcomes, all but three of the included studies compared VR with traditional physiotherapy or no intervention. Kim and Heo,^30^ Kim and Jun,^28^ and Kim et al^29^ compared two VR training programs; namely, balance training and strength training.

Overall, the qualitative findings demonstrated that VR rehabilitation programs improved gait parameters, including gait speed, cadence, step length, single support time, and single support time symmetry index.^30^ VR balance training exercises improved ankle muscle power and proprioception compared with VR strengthening exercises.^28,35^

Ankle stability and resting and walking pain also improved after implementing different VR techniques^28,32,35^; However, the improvements in foot and ankle ability measures were similar to those from traditional rehabilitation.^35^

The results from the meta-analysis showed a significant and homogeneous effect of VR on overall balance compared to those receiving conventional physiotherapy or with no intervention. It is worth mentioning that the meta-analysis was based only on four studies with moderate methodological quality. Furthermore, the subgroup analysis revealed that static balance outcomes significantly improved following VR program training. Specifically, VR training showed a significant improvement in post-intervention static balance in the medial-lateral direction.^31^ This finding can be explained by the fact that muscle strength training, sideways leg lifts, single-leg extensions, and single-leg twist exercises were performed to improve muscle strength in the medial-lateral direction while maintaining balance.^31^

Previous studies evaluating the effects of VR on ankle impairments observed inconsistent results on whether VR training was beneficial for this population. For instance, in their review, based on four studies published before 2017, Gumaa and Yousef^15^ concluded that VR is not superior to exercises. However, for this current study, we included four recent RCTs published after 2017 and provided some evidence on the effectiveness of VR for ankle joint rehabilitation. For instance, Nam et al^32^ conducted an RCT that included 28 patients with functional ankle instability, and reported a significant improvement in Limit of Stability and Cumberland ankle instability scores for the experimental group. Similarly, Kim and Heo^31^ examined the effects of a VR exercise program compared to conventional exercise on balance in a sample of patients with functional ankle instability, and found a significant difference between groups in which VR exercise was more effective in the overall direction (static) and medial-lateral direction (dynamic) of balance than conventional exercise programs.

Contrary to expectations, this study did not find a significant effect of VR training on dynamic balance. Previous literature, however, suggested a significant improvement in both static and dynamic balance of patients with ankle joint injuries after VR applications. For example, Kim and Heo^30^ compared the effectiveness of VR balance and strength programs on static and dynamic balance, and found that the two VR programs improved both outcomes. In our study, we did not observe a significant effect of VR on dynamic balance, but this observation may be due to differences in the intervention period, as a significant improvement in dynamic balance parameters may require a longer treatment period and different exercises.^31^ These inconsistencies could also be attributable to other biomechanical factors that impact dynamic balance, such as muscle strength, ankle mobility, and functionality of synovial structures that may enhance joint stiffness and thus limit dynamic balance.^36-38^ This assumption was also confirmed by Hoch et al^39^ in their investigation, where they concluded that a loss of ankle range of motion could negatively affect dynamic balance.

### Limitations

A few limitations of this systematic review should be noted. First, only full-text articles published in English were included for this analysis, and therefore we might have missed relevant studies published in other languages. Second, more than half of the included studies were published by the same author and in the same geographical location. Thus, we cannot assure that these studies used different populations. However, other articles were also included to add other critiques and viewpoints. Another methodological limitation in the study is the small individual sample sizes involved, since, with a few exceptions, most of the included studies had recruited small samples of patients that were not based on rigorous sample size calculations. Therefore, a meta-analysis was performed to account for this limitation. Lastly, the majority of the included studies received low scores on their quality assessments. However, these low scores were not because the studies were of poor quality, but rather the problem lay in the quality assessment tools. Although we attempted to use the best available quality assessment tools, these tools shared a common limitation for evaluating VR-related studies. For example, in all the included studies, blinding of participants was not possible due to the nature of VR interventions, and thus the studies missed the score for this item. This limitation was also highlighted in previous reviews,^40^ showing that there is a need to develop tools that can specifically assess VR-related studies.

### Clinical implications

The findings of this systematic review support the use of virtual reality to complement rehabilitation programs for individuals with ankle injuries, as it significantly improved gait speed, step length, single support time, cadence, and single support time symmetry index without any notable adverse events or side effects. Further, it also improved static balance and perceived ankle instability. All these outcomes are extremely important in fostering ankle joint recovery and rehabilitation after injuries.

Although it was out of the scope of this review, it is interesting to mention that VR systems may improve patient adherence. Research shows that patients’ adherence to physiotherapy is problematic and may adversely affect the success of the entire treatment plan. For example, it has been documented that nearly 10% of patients fail to attend physiotherapy sessions, about 20% do not complete their recommended exercises, and approximately 60% are completely non-adherent to prescribed physiotherapy programs.^41^

The problem of non-adherence could be minimized by including VR programs in treatment protocols, as VR has shown a high degree of enjoyment by patients.^15^ For instance, as reported by Vernadakis et al,^34^ XbK-based balance programs are more enjoyable than traditional physiotherapy programs alone. The roles of enjoyment (the desire to have fun) and intrinsic motivation (the desire to engage and expand one’s skills) have been discussed in the literature as potential predictors of non-adherence to rehabilitation interventions such as exercise.^42^ As evidenced by Vernadakis et al,^34^ VR systems do a great job in improving enjoyment, and consequently adherence to physiotherapy sessions.

Since VR systems are an emergent technology and relatively new in orthopedic rehabilitation, it is essential to train physiotherapists and clinicians on their use to achieve the desired clinical outcomes for in-patient or institution-based rehabilitation. Notably, VR treatment can also be provided as an unsupervised home-based program.^43^ To effectively use VR systems at home, patients have to be educated on how to appropriately use these technologies. This can be a challenging task, because the efficient use of VR systems depends on several factors, such as a patient’s age, technical literacy level, and previous experience.^44^ All these considerations should be addressed to achieve the optimal benefits from VR systems. It is also clear that more knowledge-to-action research is needed to effectively integrate VR applications into daily physiotherapy routines.

## Conclusion

To conclude, the existing evidence of the efficacy of VR applications for individuals with ankle joint injuries is promising. Based on this systematic review, it is valid to conclude that VR applications are feasible and comparable to other physiotherapy approaches, including exercise, and thus can be considered as a realistic alternative for the rehabilitation of patients with ankle injuries. The results from the meta-analysis of four clinical trials showed a significant and homogeneous effect of VR on static balance compared to conventional or no intervention. For dynamic balance, however, the evidence of the effectiveness of VR applications is somewhat inconclusive. A point of note is that the quality of the reviewed studies must be carefully considered while assessing the results of this systematic review because only two studies were deemed to have good quality, while the quality of the rest varied from poor to fair. Also, only three small studies were included in each meta-analysis, which may imply low power and limited generalizability. In order to effectively inform clinical practice, further clinical trials with larger sample sizes are needed.

## Supplemental Material

sj-docx-1-rpo-10.1177_11795727231151636 – Supplemental material for The Efficacy of Virtual Reality Training in the Rehabilitation of Orthopedic Ankle Injuries: A Systematic Review and Meta-analysis Click here for additional data file. Supplemental material, sj-docx-1-rpo-10.1177_11795727231151636 for The Efficacy of Virtual Reality Training in the Rehabilitation of Orthopedic Ankle Injuries: A Systematic Review and Meta-analysis by Abd El Rahman Elaraby, Mostafa Shahien, Alhadi M. Jahan, Mohammad Etoom and Amira Hassan Bekhet in Advances in Rehabilitation Science and Practice

sj-docx-2-rpo-10.1177_11795727231151636 – Supplemental material for The Efficacy of Virtual Reality Training in the Rehabilitation of Orthopedic Ankle Injuries: A Systematic Review and Meta-analysis Click here for additional data file. Supplemental material, sj-docx-2-rpo-10.1177_11795727231151636 for The Efficacy of Virtual Reality Training in the Rehabilitation of Orthopedic Ankle Injuries: A Systematic Review and Meta-analysis by Abd El Rahman Elaraby, Mostafa Shahien, Alhadi M. Jahan, Mohammad Etoom and Amira Hassan Bekhet in Advances in Rehabilitation Science and Practice

## References

[1] Schiftan GS Ross LA Hahne AJ. The effectiveness of proprioceptive training in preventing ankle sprains in sporting populations: a systematic review and meta-analysis. J Sci Med Sport. 2015;18:238-244. 24831756 10.1016/j.jsams.2014.04.005

[2] Waterman BR Owens BD Davey S Zacchilli MA Belmont PJ. The epidemiology of ankle sprains in the United States. J Bone Joint Surg. 2010;92:2279-2284. 20926721 10.2106/JBJS.I.01537

[3] Cooke MW. A survey of current consultant practice of treatment of severe ankle sprains in emergency departments in the United Kingdom. Emerg Med J. 2003;20:505-507. 14623832 10.1136/emj.20.6.505 PMC1726246

[4] Hung YJ. Neuromuscular control and rehabilitation of the unstable ankle. World J Orthop. 2015;6:434-438. 26085985 10.5312/wjo.v6.i5.434 PMC4458494

[5] Thompson C Schabrun S Romero R Bialocerkowski A van Dieen J Marshall P. Factors contributing to chronic ankle instability: a systematic review and meta-analysis of systematic reviews. Sports Med. 2018;48:189-205. 28887759 10.1007/s40279-017-0781-4

[6] Lu A Wang X Huang D , et al. The effectiveness of lateral ankle ligament reconstruction when treating chronic ankle instability: a systematic review and meta-analysis. Injury. 2020;51:1726-1732. 32534817 10.1016/j.injury.2020.05.031

[7] Kaminski TW Hartsell HD. Factors contributing to chronic ankle instability: a strength perspective. J Athl Train. 2002;37:394-405. 12937561 PMC164371

[8] Yeung MS Chan KM So CH Yuan WY. An epidemiological survey on ankle sprain. Br J Sports Med. 1994;28:112-116. 7921910 10.1136/bjsm.28.2.112 PMC1332043

[9] Valderrabano V Hintermann B Dick W. Scandinavian total ankle replacement: a 3.7-year average followup of 65 patients. Clin Orthop Relat Res. 2004;424:47-56. 15241143

[10] National Institute for Health and Care Excellence. Fractures (Non-Complex): Assessment and Management. 2016. Accessed November 9, 2022. https://www.nice.org.uk/guidance/ng38 26913311

[11] Balduini FC Vegso JJ Torg JS Torg E. Management and rehabilitation of ligamentous injuries to the ankle. Sports Med. 1987;4:364-380. 3313619 10.2165/00007256-198704050-00004

[12] Vernadakis N Gioftsidou A Antoniou P Ioannidis D Giannousi M. The impact of Nintendo Wii to physical education students’ balance compared to the traditional approaches. Comput Educ. 2012;59:196-205.

[13] Cipresso P Giglioli IAC Raya MA Riva G. The past, present, and future of virtual and augmented reality research: a network and cluster analysis of the literature. Front Psychol. 2018;9:2086. 30459681 10.3389/fpsyg.2018.02086 PMC6232426

[14] Mazurek J Kiper P Cieślik B , et al. Virtual reality in medicine: a brief overview and future research directions. Hum Mov. 2019;20:16-22.

[15] Gumaa M Rehan Youssef A. Is virtual reality effective in orthopedic rehabilitation? A systematic review and meta-analysis. Phys Ther. 2019;99:1304-1325. 31343702 10.1093/ptj/pzz093

[16] Corbetta D Imeri F Gatti R. Rehabilitation that incorporates virtual reality is more effective than standard rehabilitation for improving walking speed, balance and mobility after stroke: a systematic review. J Physiother. 2015;61:117-124. 26093805 10.1016/j.jphys.2015.05.017

[17] Laver KE Lange B George S Deutsch JE Saposnik G Crotty M. Virtual reality for stroke rehabilitation. Cochrane Database Syst Rev. 2017;11:CD008349. 10.1002/14651858.CD008349.pub4 PMC6485957 29156493

[bibr18-11795727231151636] Dockx K Bekkers EM Van den Bergh V , et al. Virtual reality for rehabilitation in Parkinson’s disease. Cochrane Database Syst Rev. 2016;12:CD010760. 10.1002/14651858.CD010760.pub2 PMC6463967 28000926

[19] Massetti T Trevizan IL Arab C Favero FM Ribeiro-Papa DC de Mello Monteiro CB. Virtual reality in multiple sclerosis: a systematic review. Mult Scler Relat Disord. 2016;8:107-112. 27456884 10.1016/j.msard.2016.05.014

[20] Page MJ McKenzie JE Bossuyt PM , et al. The PRISMA 2020 statement: an updated guideline for reporting systematic reviews. BMJ. 2021;372:n71. 10.1136/bmj.n71 PMC8005924 33782057

[21] Downs SH Black N. The feasibility of creating a checklist for the assessment of the methodological quality both of randomised and non-randomised studies of health care interventions. J Epidemiol Community Health. 1998;52:377-384. 9764259 10.1136/jech.52.6.377 PMC1756728

[22] Cohen J. Statistical Power Analysis for the Behavioral Sciences. 2nd ed. Routledge; 1988.

[23] Sims J Cosby N Saliba EN Hertel J Saliba SA. Exergaming and static postural control in individuals with a history of lower limb injury. J Athl Train. 2013;48:314-325. 23675790 10.4085/1062-6050-48.2.04 PMC3655744

[24] McPhail SM O’Hara M Gane E Tonks P Bullock-Saxton J Kuys SS. Nintendo Wii fit as an adjunct to physiotherapy following lower limb fractures: preliminary feasibility, safety and sample size considerations. Physiotherapy. 2016;102:217-220. 26209909 10.1016/j.physio.2015.04.006

[25] Mohammadi N Hadian MR Olyaei G. The Effects of Wii fit plus training on functional ability in athletes with functional ankle instability. Sports Orthop Traumatol. 2020;36:52-59.

[26] Kim K Choi B Lim W. The efficacy of virtual reality assisted versus traditional rehabilitation intervention on individuals with functional ankle instability: a pilot randomized controlled trial. Disabil Rehabil Assist Technol. 2019;14:276-280. 29385840 10.1080/17483107.2018.1429501

[bibr27-11795727231151636] Kim KJ Gang MY. Effect of taping and virtual reality combined exercise on static and dynamic balance with functional ankle instability. Phys Ther Korea. 2020;27:292-297.

[bibr28-11795727231151636] Kim KJ Jun HJ. Effects of virtual reality programs on proprioception and instability of functional ankle instability. J Int Acad Phys Ther Res. 2015;6:891-895. 10.1589/jpts.27.3097 PMC4668143 26644652

[bibr29-11795727231151636] Kim KJ Jun HJ Heo M. Effects of Nintendo Wii fit plus training on ankle strength with functional ankle instability. J Phys Ther Sci. 2015;27:3381-3385. 26696703 10.1589/jpts.27.3381 PMC4681910

[bibr30-11795727231151636] Kim KJ Heo M. Effects of virtual reality programs on balance in functional ankle instability. J Phys Ther Sci. 2015;27:3097-3101. 26644652 10.1589/jpts.27.3097 PMC4668143

[bibr31-11795727231151636] Kim KJ Heo M. Comparison of virtual reality exercise versus conventional exercise on balance in patients with functional ankle instability: a randomized controlled trial. BMR. 2019;32:905-911. 10.3233/BMR-181376 30958334

[bibr32-11795727231151636] Nam SM Kim K Lee DY. Effects of visual feedback balance training on the balance and ankle instability in adult men with functional ankle instability. J Phys Ther Sci. 2018;30:113-115. 29410578 10.1589/jpts.30.113 PMC5788787

[bibr33-11795727231151636] Punt IM Armand S Ziltener JL Allet L. Effect of Wii Fit^TM^ exercise therapy on gait parameters in ankle sprain patients: a randomized controlled trial. Gait Posture. 2017;58:52-58. 28735202 10.1016/j.gaitpost.2017.06.284

[bibr34-11795727231151636] Vernadakis N Derri V Tsitskari E Antoniou P. The effect of Xbox Kinect intervention on balance ability for previously injured young competitive male athletes: a preliminary study. Phys Ther Sport. 2014;15:148-155. 24239167 10.1016/j.ptsp.2013.08.004

[35] Punt IM Ziltener JL Monnin D Allet L. Wii Fit^TM^ exercise therapy for the rehabilitation of ankle sprains: its effect compared with physical therapy or no functional exercises at all. Scand J Med Sci Sports. 2016;26:816-823. 26076737 10.1111/sms.12509

[36] Runge CF Shupert CL Horak FB Zajac FE. Ankle and hip postural strategies defined by joint torques. Gait Posture. 1999;10:161-170. 10502650 10.1016/s0966-6362(99)00032-6

[bibr37-11795727231151636] Horak FB. Postural orientation and equilibrium: what do we need to know about neural control of balance to prevent falls? Age Ageing. 2006;35:ii7-ii11. 10.1093/ageing/afl077 16926210

[38] Hernández-Guillén D Tolsada-Velasco C Roig-Casasús S Costa-Moreno E Borja-de-Fuentes I Blasco JM. Association ankle function and balance in community-dwelling older adults. PLoS One. 2021;16:e0247885. 10.1371/journal.pone.0247885 PMC7932177 33661991

[39] Hoch MC Staton GS McKeon PO. Dorsiflexion range of motion significantly influences dynamic balance. J Sci Med Sport. 2011;14:90-92. 20843744 10.1016/j.jsams.2010.08.001

[40] Asadzadeh A Samad-Soltani T Salahzadeh Z Rezaei-Hachesu P. Effectiveness of virtual reality-based exercise therapy in rehabilitation: a scoping review. Inform Med Unlocked. 2021;24:100562.

[41] Bassett SF Prapavessis H. A test of an adherence-enhancing adjunct to physiotherapy steeped in the protection motivation theory. Physiother Theor Pract. 2011;27:360-372. 10.3109/09593985.2010.507238 20795875

[42] Lakicevic N Gentile A Mehrabi S , et al. Make fitness fun: could novelty be the key determinant for physical activity adherence? Front Psychol. 2020;11:577522. 33178079 10.3389/fpsyg.2020.577522 PMC7593334

[43] Tokgöz P Stampa S Wähnert D Vordemvenne T Dockweiler C. Virtual reality in the rehabilitation of patients with injuries and diseases of upper extremities. Healthcare. 2022;10:1124. 35742176 10.3390/healthcare10061124 PMC9222955

[44] Halbig A Babu SK Gatter S Latoschik ME Brukamp K von Mammen S. Opportunities and challenges of virtual reality in healthcare: a domain experts inquiry. Front Virtual Real. 2022;3:837616.

