# Supplementary material for: The Efficacy of Virtual Reality Training in the Rehabilitation of Orthopedic Ankle Injuries: A Systematic Review and Meta-analysis
Source: Adv Rehabil Sci Pract. 2023 Feb 7;12:11795727231151636. doi: 10.1177/11795727231151636 (PMC9933927; doi:10.1177/11795727231151636)
Supplement: sj-docx-2-rpo-10.1177_11795727231151636 – Supplemental material for The Efficacy of Virtual Reality Training in the Rehabilitation of Orthopedic Ankle Injuries: A Systematic Review and Meta-analysis [file sj-docx-2-rpo-10.1177_11795727231151636.docx]

| Quality Assessment items | Kim and Heo ^31^ | Punt et al.^33^ | Kim et al.^26^ | Kim & Jun^28^ | Punt et al. ^35^ | Nam et al. ^32^ | Kim & Heo ^30^ | Vernadakis et al. ^34^ | Kim & Gang ^27^ | Kim et al. ^29^ |
| --- | --- | --- | --- | --- | --- | --- | --- | --- | --- | --- |
| Reporting sum score | 9 | 9 | 8 | 8 | 9 | 7 | 7 | 7 | 8 | 8 |
| Is the hypothesis/aim/objective of the study clearly described? | 1 | 1 | 1 | 1 | 1 | 1 | 1 | 1 | 1 | 1 |
| Are the main outcomes to be measured clearly described in the introduction or methods section? | 1 | 1 | 1 | 1 | 1 | 1 | 1 | 1 | 1 | 1 |
| Are the characteristics of the patients included in the study clearly described? | 1 | 1 | 1 | 1 | 1 | 1 | 1 | 1 | 1 | 1 |
| Are the interventions of interest clearly described? | 1 | 1 | 1 | 1 | 1 | 1 | 1 | 1 | 1 | 1 |
| Are the distributions of principal confounders in each group of patients to be compared clearly described? | 1 | 1 | 1 | 1 | 1 | 1 | 1 | 1 | 1 | 1 |
| Are the main findings of the study clearly described? | 1 | 1 | 1 | 1 | 1 | 1 | 1 | 1 | 1 | 1 |
| Does the study provide estimates of the random variability in the data for the main outcomes? | 1 | 1 | 1 | 1 | 1 | 1 | 1 | 1 | 1 | 1 |
| Have all important adverse events that may be a consequence of the intervention been reported? | 0 | 0 | 0 | 0 | 0 | 0 | 0 | 0 | 0 | 0 |
| Have the characteristics of patients lost to follow-up been described? | 1 | 1 | 0 | 0 | 1 | 0 | 0 | 0 | 0 | 0 |
| Have actual probability values been reported (e.g., 0.035 rather than<0.05) for the main outcomes, except where the probability value is  <0.001? | 1 | 1 | 1 | 1 | 1 | 0 | 0 | 0 | 1 | 1 |
| External validity sum score | 0 | 0 | 0 | 0 | 0 | 0 | 0 | 0 | 0 | 0 |
| Were the subjects asked to participate in the study representative of the entire population from which they were recruited? | 0 | 0 | 0 | 0 | 0 | 0 | 0 | 0 | 0 | 0 |
| Were those subjects who were prepared to participate representative of the entire population from which they were recruited? | 0 | 0 | 0 | 0 | 0 | 0 | 0 | 0 | 0 | 0 |
| Were the staff, places, and facilities where the patients were treated representative of the treatment the majority of patients receive? | 0 | 0 | 0 | 0 | 0 | 0 | 0 | 0 | 0 | 0 |
| Internal validity sum score | 8 | 10 | 5 | 7 | 10 | 6 | 7 | 7 | 7 | 7 |
| Was an attempt made to blind study subjects to the intervention they have received? | 0 | 0 | 0 | 0 | 0 | 0 | 0 | 0 | 0 | 0 |
| Was an attempt made to blind those measuring the main outcomes of the intervention? | 0 | 1 | 0 | 0 | 1 | 0 | 0 | 0 | 0 | 0 |
| If any of the results of the study were based on “data dredging,” was this made clear? | 1 | 1 | 1 | 1 | 1 | 1 | 1 | 1 | 1 | 1 |
| In trials and cohort studies, do the analyses adjust for different lengths of follow-up of patients, or in case-control studies, is the time period between the intervention and outcome the same for cases and controls? | 1 | 1 | 1 | 0 | 1 | 1 | 1 | 1 | 1 | 1 |
| Were the statistical tests used to assess the main outcomes appropriate? | 1 | 1 | 1 | 1 | 1 | 1 | 1 | 1 | 1 | 1 |
| Was compliance with the intervention(s) reliable? | 1 | 1 | 1 | 1 | 1 | 1 | 1 | 1 | 1 | 1 |
| Were the main outcome measures used accurate (valid and reliable)? | 1 | 1 | 1 | 1 | 1 | 1 | 1 | 1 | 1 | 1 |
| Were the patients in different intervention groups (trials and cohort studies) or were the cases and controls (case-control studies) recruited from the same population? | 1 | 1 | 1 | 0 | 1 | 0 | 1 | 1 | 1 | 1 |
| Were study subjects in different intervention groups (trials and cohort studies) or were the cases and controls (case-control studies) recruited over the same period of time? | 0 | 0 | 0 | 0 | 0 | 0 | 0 | 0 | 0 | 0 |
| Were study subjects randomized to intervention groups? | 1 | 1 | 1 | 1 | 1 | 1 | 1 | 1 | 1 | 1 |
| Was the randomized intervention assignment concealed from both patients and healthcare staff until recruitment was complete and irrevocable? | 0 | 0 | 0 | 0 | 0 | 0 | 0 | 0 | 0 | 0 |
| Was there adequate adjustment for confounding in the analyses from which the main findings were drawn? | 0 | 0 | 0 | 0 | 1 | 0 | 0 | 0 | 0 | 0 |
| Were losses of patients to follow-up taken into account? | 1 | 1 | 0 | 0 | 1 | 0 | 0 | 0 | 0 | 0 |
| Power | 0 | 1 | 0 | 0 | 1 | 0 | 0 | 0 | 0 | 0 |
| Did the study have sufficient power to detect a clinically important effect where the probability value for a difference being due to chance is less than 5%? Sample sizes have been calculated to detect a difference of x% and y%. | 0 | 1 | 0 | 0 | 1 | 0 | 0 | 0 | 0 | 0 |
| Final score | 17 | 20 | 15 | 13 | 20 | 13 | 14 | 14 | 15 | 15 |
